# Time on timing: Dissociating premature responding from interval sensitivity in Parkinson's disease

**DOI:** 10.1002/mds.26631

**Published:** 2016-04-19

**Authors:** Jiaxiang Zhang, Cristina Nombela, Noham Wolpe, Roger A. Barker, James B. Rowe

**Affiliations:** ^1^ Cardiff University Brain Research Imaging CentreSchool of PsychologyCardiff UniversityCardiffUK; ^2^ Cognition and Brain Sciences UnitMedical Research CouncilCambridgeUK; ^3^ Department of Clinical NeurosciencesUniversity of CambridgeCambridgeUK

**Keywords:** Parkinson's disease, temporal bisection, temporal trisection, response time, computational modeling

## Abstract

**Background:**

Parkinson's disease (PD) can cause impulsivity with premature responses, but there are several potential mechanisms. We proposed a distinction between poor decision‐making and the distortion of temporal perception. Both effects may be present and interact, but with different clinical and pharmacological correlates.

**Objectives:**

This study assessed premature responding during time perception in PD.

**Methods:**

In this study, 18 PD patients and 19 age‐matched controls completed 2 temporal discrimination tasks (bisection and trisection) and a baseline reaction‐time task. Timing sensitivity and decision‐making processes were quantified by response and response time. An extended version of the modified difference model was used to examine the precision of time representation and the modulation of response time by stimulus ambiguity.

**Results:**

In the bisection task, patients had a lower bisection point (*P* < .05) and reduced timing sensitivity when compared with controls (*P* < .001). In the trisection task, patients showed lower sensitivity in discriminating between short and medium standards (*P* < .05). The impairment in timing sensitivity correlated positively with patients' levodopa dose equivalent (*P* < .05). Critically, patients had disproportionately faster response times when compared with controls in more ambiguous conditions, and the degree of acceleration of response time increased with disease severity (*P* < .05). Computational modeling indicated that patients had poorer precision in time representation and stronger modulation of response time by task ambiguity, leading to smaller scaling of the decision latency (*P* < .05).

**Conclusions:**

These findings suggest that timing deficits in PD cannot be solely attributed to perceptual distortions, but are also associated with impulsive decision strategies that bias patients toward premature responses. © 2016 The Authors. Movement Disorders published by Wiley Periodicals, Inc. on behalf of International Parkinson and Movement Disorder Society

Impulsivity is a common nonmotor symptom of Parkinson's disease (PD), even in patients without clinically diagnosed impulsive control disorders.^1^, ^2^, ^3^ Impulsive decisions refer to premature or inappropriate acts as a result of poor evaluation of information, reward, risk, or cost.^4^ They can be manifested in various ways in PD, such as deficits in reward processing,^5^ risk seeking,^6^, ^7^ and impatience.^8^ Some of the impulsive acts can be worsened by normal dopaminergic remediation,^9^ possibly because of relative dopamine‐overdose in the ventral striatum and mesocortical pathways.^10^

Recent studies have led to the hypothesis that distortion in time perception is also a factor underlying impulsivity.^9^, ^11^ For example, delay‐intolerance and accelerated temporal discounting in PD^12^, ^13^ might be explained by an overestimation of the time interval between actions and outcomes, which leads to devaluing temporally delayed rewards.^14^ This introduces a potential link between impulsive decision‐making and timing deficits in PD^15^, ^16^ and calls for a better understanding of the effect of the disease on time processing.

Understanding the pathophysiological timing deficits in PD is important for several other reasons. First, parkinsonian bradykinesia,^17^, ^18^ which includes a deficit of rhythmic tapping^19^, ^20^ and prolonged response time in sequential movements,^21^ has been linked to a deficit in the perception or reproduction of supra‐second intervals in the motor domain.^15^ Moreover, dopaminergic dysfunction is associated with the altered speed of the “internal clock.”^22^, ^23^ This is supported by both humans and animal models showing that dopamine antagonists and agonists can differentially increase or decrease the clock speed,^24^, ^25^, ^26^ leading to impaired performance in the perception of time.^27^, ^28^, ^29^ Finally, PD affects an extensive brain network that is involved in the perception of time intervals from milliseconds to seconds,^30^ including the caudate and putamen, the prefrontal cortex, and the cerebellum.^31^, ^32^, ^33^, ^34^

Although decision‐making and time perception are both abnormal in PD, it is yet unknown whether impaired decision‐making has contributed to the observed timing deficits. For most behaviors involving accurate timing, a decision process is required to read out the timing information and generate corresponding responses.^22^, ^23^

The present study assessed premature responding during the perception of time in PD. We compared the performance of patients with PD with that of healthy controls, first in a classical temporal bisection task^35^ and then in a temporal trisection task. The two tasks shared similar perceptual information (subsecond intervals) but varied in their cognitive loads and decision rules (binary or ternary choices) to examine the generalizability of the effects.

Notably, an inference on impulsivity together with timing deficits within the same task was made possible by quantifying not only patients' choices but also their response time (RT). Because more difficult decisions are typically associated with longer RT (eg, the conflict‐induced slowing effect^36^), the RT provided an index of the decision process,^37^ which is largely neglected in previous studies of interval timing.

We showed that in ambiguous or high‐conflict conditions, PD patients would make disproportionately faster decisions: a signature of premature responding.^38^ We then used an extended version of the modified difference model^35^ to account for responses and RTs in time perception. The model decomposed behavioral performance into parameter estimates of precision of time representation and decision latency. An inference on model parameters allowed us to further dissociate premature responding from internal clock impairments in PD.

## Methods

### Participants

A total of 18 patients with idiopathic PD were recruited from the PD research clinic at the John Van Geest Centre for Brain Repair. Inclusion criteria were clinically diagnosed idiopathic PD according to the UK PD brain bank criteria,^39^ in early to mid‐stage of the disease with Hoehn and Yahr stages 1 to 3,^40^ on dopaminergic medication, and not demented based on prior cognitive assessment. Cognitive abilities in both patients and controls were reassessed through the Mini Mental State Examination^41^ and Addenbrooke's Cognitive Examination Revised,^42^ which is sensitive to mild cognitive decline in PD.^43^ Only patients with Addenbrooke's Cognitive Examination Revised above 84/100 were included in the study. All of the patients were on levodopa therapy at the time of the experiment (drug details are summarized in Supplementary Table 1). Levodopa equivalent daily dose (LEDD) was computed to facilitate a comparison between all of the patients.^44^, ^45^ The severity of symptoms in patients was assessed with the Unified Parkinson's Disease Rating Scale.^46^ See Table 1 for demographic and neuropsychological information.

**Table 1:** Demographic details of Parkinson's patients and controls

	Patients, n = 18	Controls, n = 19	Statistic, *P* value
Male/female	12/6	11/8	.58
Age	69.18 (48‐81)	67.47 (55‐76)	.58
Disease duration, y	12.48 (6‐26)	–	–
MMSE	28.28 (23‐30)	29.84 (29‐30)	.0001
ACE‐R	91.72 (84‐98)	97.26 (91‐100)	.001
UPDRS	33.5 (23‐51)	–	–
LEDD, mg/day	1446 (640‐2610)	–	–

Group difference was evaluated by χ^2^ test (for gender) or *t*‐test (for age, MMSE, and Addenbrooke's Cognitive Examination). ACE‐R, Addenbrooke's Cognitive Examination Revised; LEDD, levodopa equivalent daily dose.

A total of 19 age‐ and sex‐matched healthy control participants were recruited with no significant neurological or psychiatric history. All of the participants gave written informed consent. The study was approved by the Cambridge Research Ethics Committee.

### Task and Procedure

Each participant performed two time perception tasks: temporal bisection and temporal trisection. The order of the tasks was randomized across participants. Before the first timing task, the participants performed a baseline‐reaction‐time task.

#### Temporal Bisection Task

**Figure 1:**
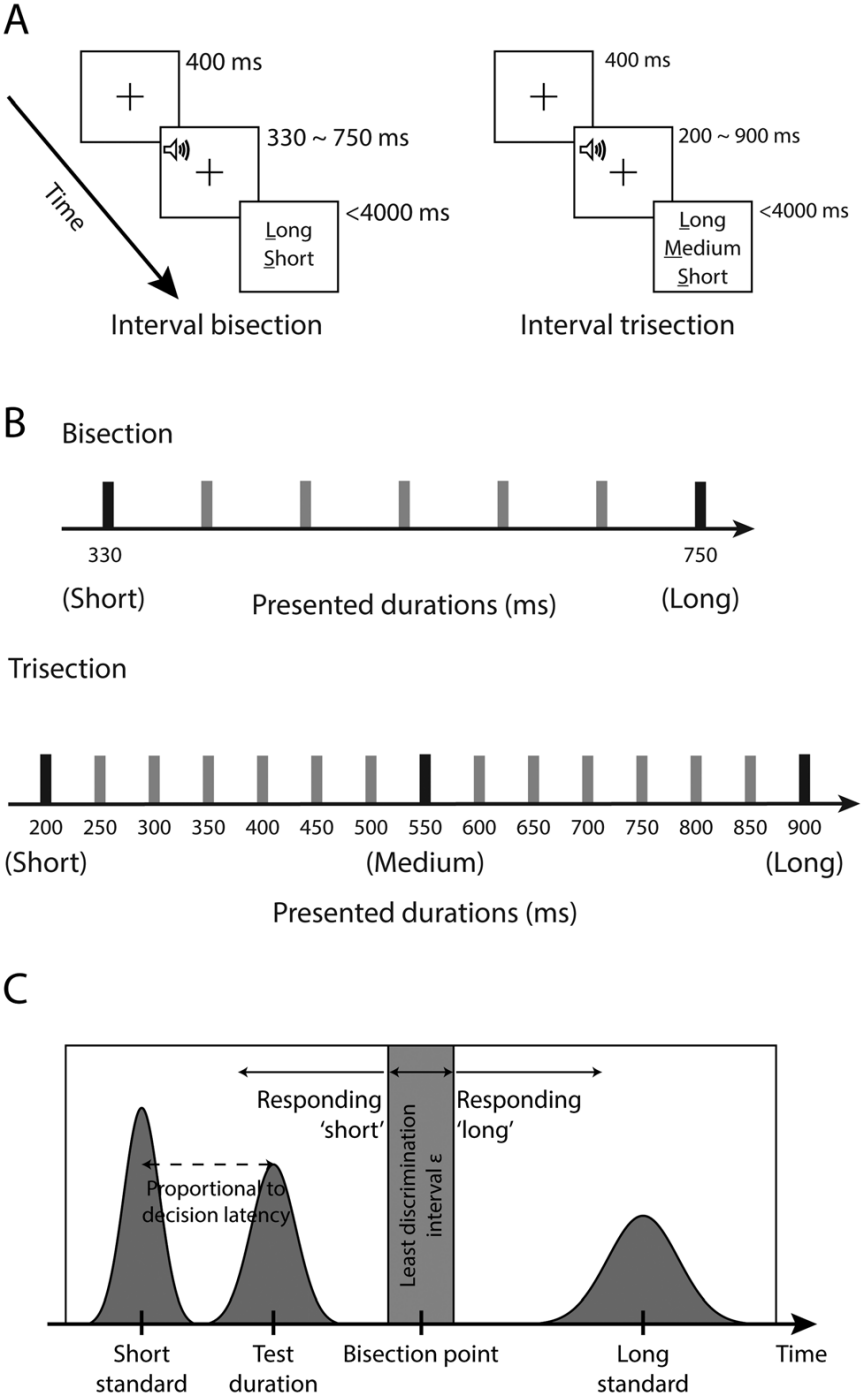
A: Structure of a single trial in the temporal bisection (*left*) and temporal trisection (*right*) tasks. **B**: The presented durations in the bisection and trisection tasks. Participants were familiarized with the standards before and during the experiments. **C**: Computational model for temporal discrimination. The model assumes that on each trial, the subjective perception of the duration standards and the test duration are sampled from normal distributions with means equal to the true physical duration and variances proportional to the means (scaled by *σ*). If the bisection point is *B*, the model predicts a “short” response when the test duration is smaller than *B*‐*ε*/2, and a “long” response when the duration is larger than *B* + *ε*/2. For a test duration between [*B*‐*ε*/2, *B* + *ε*/2], the model predicts a random response. The model prediction of decision latency is a function of the distance between the test duration and the nearest standard.

In each trial, participants were instructed to decide whether the duration of an auditory tone was more similar to a short (330 milliseconds) or a long standard (750 milliseconds). A total of 7 arithmetically spaced durations were presented (Fig. 1A,B).

After a short practice, the participants performed 2 blocks of 35 trials, during which each duration was presented in 10 trials in a pseudo‐randomized order. Before each block, the participants were familiarized with the short and long standards in 4 presentations.

In each trial, a central fixation was displayed for 400 milliseconds, followed by an auditory tone with one of the 7 durations (see Supplementation Methods). After tone offset, the participants were asked to press one of the two buttons based on their perceived tone duration in comparison to the two standards. The participants had a maximum of 4000 milliseconds to make a response. The intertrial interval was randomized between 1500 milliseconds and 2100 milliseconds. The response mappings for the short and long decisions were counterbalanced across participants.

#### Temporal Trisection Task

The participants were required to decide whether a time interval presented on each trial is more similar to short (200 milliseconds), medium (550 milliseconds), or long standards (900 milliseconds). A total of 15 durations were presented (Fig. 1B). Task procedure and trial structure are similar to that of the bisection task. The task was composed of 4 blocks of 45 trials, during which each of the 15 durations was presented in 12 trials in a pseudo‐randomized order. Before each block, the participants were exposed to 4 presentations of each of the three standards. The response mappings for the short and long decisions were counterbalanced across participants.

#### Baseline‐Reaction‐Time Task

The task was composed of 50 trials. On each trial, participants pressed a button with their right index finger as soon as a visual response cue appeared. The cue was presented for a maximum of 3000 milliseconds. The intertrial interval was randomized between 1200 milliseconds and 6200 milliseconds.

### Data Analysis

For the bisection task, we calculated the proportion of long responses for each of the tested durations. Following the method used in previous studies,^26^, ^35^ a linear regression was calculated from the steepest part of the psychometric function (from 400 milliseconds to 680 milliseconds). The slope and intercept from the regression line were used to estimate 2 measures that quantified response bias and timing sensitivity in individual participants: bisection point (BP) and Weber ratio (WR). The BP is the 50% response threshold from the psychometric function, which reflects the temporal duration at which the participant made a 50% long response. The WR is the ratio between half the distance between the 75% and 25% thresholds and the BP value, which is a normalized index of temporal sensitivity. A smaller WR indicates steeper psychometric function slopes and therefore more sensitive timing.

For the trisection task, the psychometric function was generated by calculating the proportion of medium responses. Two sets of BP and WR values were calculated: one for the short‐to‐medium duration range and one for the medium‐to‐long duration range.

In both the bisection and trisection tasks, a participant's RT was measured as the latency between tone offset and response. Chronometric functions were obtained by calculating the mean RT at each stimulus duration.

### Computational Modeling

An extended version of the modified difference model of time perception^35^ was developed to fit individual participants' behavioral data (Fig. 1C). A total of 4 variants of the model were considered. The best‐fit model assumed the scalar property and nonlinear, nondecision latencies (see Supplementary Methods and Supplementary Fig. 2).

**Figure 2:**
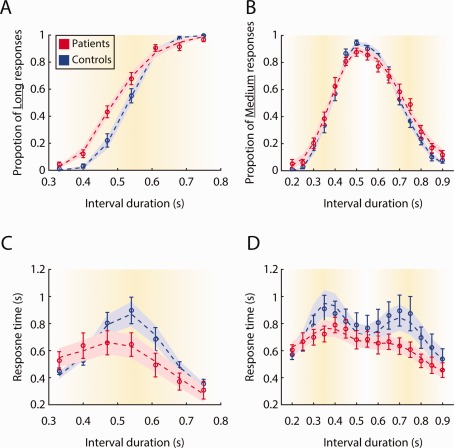
The behavioral responses of Parkinson's disease patients and controls in temporal discrimination tasks. **A**: Proportion of long responses in the bisection task. **B**: Proportion of medium responses in the trisection task. **C**: Mean response time in the bisection task. **D**: Mean response time in the trisection task. Error bars denote standard errors of the mean across participants. Dashed lines and shaded areas denote model‐predicted means and standard errors, which were obtained by averaging the results from 100 simulations with best‐fitted model parameters for each participant. [Color figure can be viewed in the online issue, which is available at wileyonlinelibrary.com.]

The model decomposed behavioral data into several parameters that mapped onto cognitive processes during time perception (see Supplementary Methods for details and model fitting procedure). The coefficient of variation *σ* describes the precision of subjective sense of a time interval (large *σ* refers to low precision). Least discriminable interval *ε* describes the minimum time interval around the bisection point, within which the model would predict a random response. Bisection point bias *θ* describes the deviation of the bisection point from the arithmetic mean of 2 standard intervals. The *s*caling of decision latency *a* describes how decision latencies scale with interval ambiguity (large *a* refers to a large RT in ambiguous conditions). Nondecision latency *b* describes the maximum delay of stimulus encoding and motor response.^47^ Parameters *c* and *d* constrain the nonlinear relationship between the nondecision latency and the presented duration.

## Results

### Temporal Bisection and Trisection Responses

When standard durations were presented, PD patients and controls had high decision accuracy in the bisection task (Fig. 2A, short standard accuracy: patients, 95.56% ± 1.66% standard error of the mean [SEM] and controls, 98.95% ± 1.05%; long standard: patients, 96.53% ± 1.45% and controls, 99.47% ± 0.53%) and the trisection task (Fig. 2B, short standard: patients, 93.98% ± 2.92% and controls, 98.68% ± 0.96%; medium standard: patients, 85.48% ± 3.36% and controls, 90.23% ± 1.96%; long standard: patients, 87.25% ± 3.04% and controls, 92.11% ± 2.06%).

For the bisection task, a repeated‐measures analysis of variance (ANOVA) on bisection responses (proportion of long responses) showed significant main effects of group (patient or control, *F*_1,35_ = 5.78, *P* < .05) and durations (*F*_6,210_ = 436.85, *P* < .0001), and a significant group by duration interaction (*F*_6,210_ = 5.67, *P* < .0001), indicating that patients responded differently when compared with controls. PD patients had a smaller bisection point (BP) (*P* < .05, permutation test) and a larger Weber ratio (WR) than controls (*P* < .001), suggesting that PD patients had a bisection bias toward shorter durations and impaired discriminability of temporal intervals (Supplementary Table 2).

For the trisection task, a repeated‐measures ANOVA on trisection responses (proportion of medium responses) showed a significant main effect of duration (*F*_14,490_ = 183.84, *P* < .0001), but no effect of group (*F*_1,35_ = 0.88, *P* = .35) nor group by duration interaction (*F*_14,490_ = 1.44, *P* = .13). Two sets of BP and WR values were calculated: one based on the durations between short and medium standards and the other based on the durations between medium and long standards (Supplementary Table 1). There was no significant difference in BP values between groups (short‐medium durations: *P* = .83; medium‐long durations: *P* = .48, permutation test), indicating that patients' response bias in the bisection task did not generalize to the trisection task. WR in PDs was larger than that in controls in the short‐medium duration range (*P* < .05, permutation test), but WR did not differ significantly between groups in the medium‐long duration range (*P* = .23).

**Figure 3:**
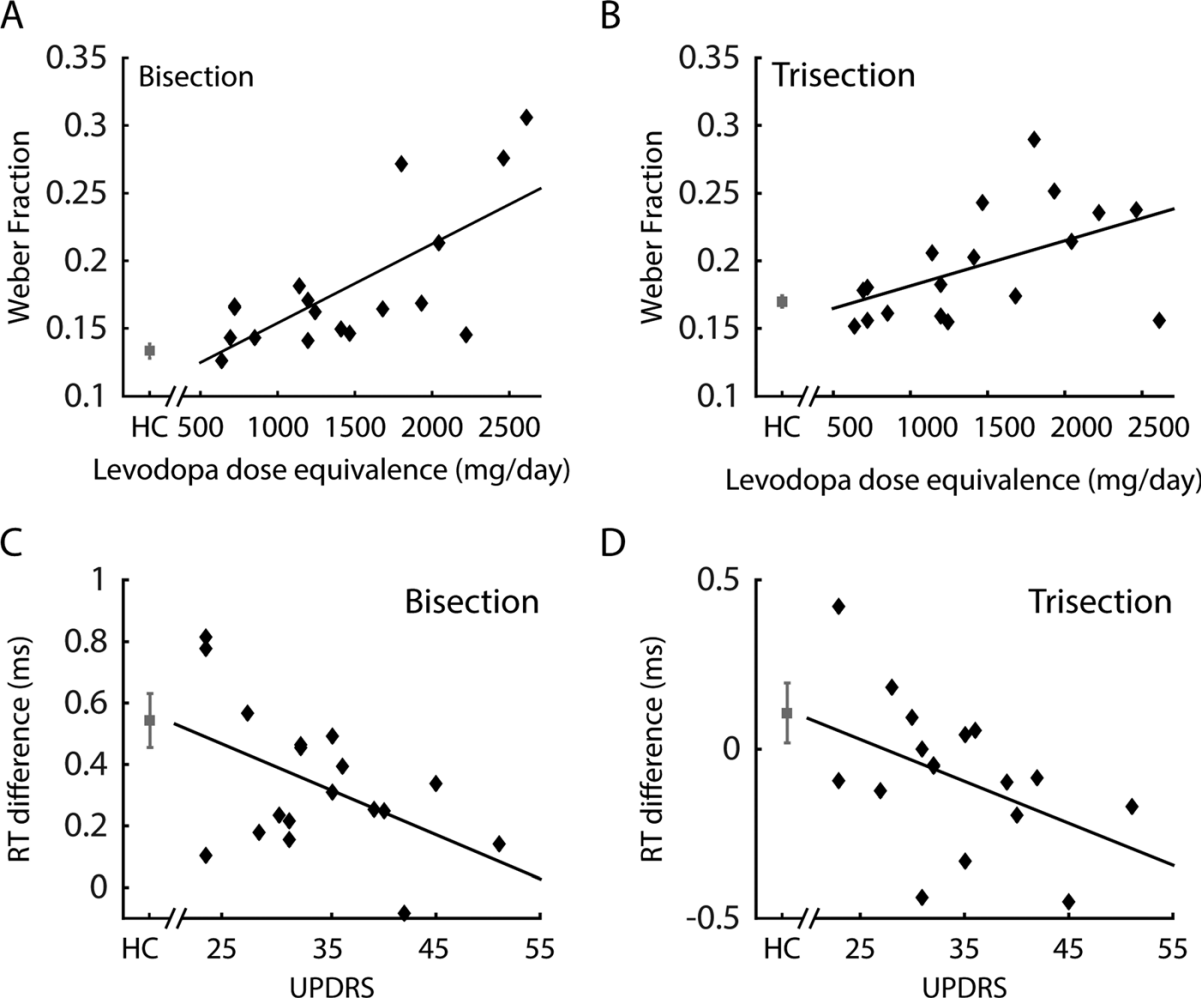
Correlations between the Weber ratio and levodopa equivalent daily dose in PD patients in the bisection task (**A**) and the trisection task in the short‐medium range (**B**). **C**: Correlation between the UPDRS and response time difference in the bisection task. **D**: Correlation between the UPDRS and response time difference in the trisection task. Grey data points and error bars: means and standard errors of controls.

Further correlational analyses yielded a significant positive correlation between LEDD and WR in both the bisection (*R* = .70, *P* < .01) and trisection (short‐medium range, *R* = .50, *P* < .05) tasks in patients, indicating that PD patients with higher daily doses of dopaminergic medication show more pronounced impairment (Fig. 3A,B). There was no significant correlation between LEDD and the trisection WR in the medium‐long range (*R* = .11, *P* = .67).

### Response Time on Temporal Bisection and Trisection

For the bisection task, a repeated‐measures ANOVA on RT showed a significant main effect of duration (*F*_6,210_ = 30.83, *P* < .0001), confirming that the RT was modulated by duration of presentation (Fig. 2C). RT is longer when the presented duration is further away from the two standards, that is, participants slow down their responses when durations are more ambiguous for bisection. There was no significant main effect of group (*F*_1,35_ = 0.79, *P* = .38), but there was a significant duration by group interaction (*F*_6,210_ = 5.54, *P* < .0001).

Chronometric functions revealed that this interaction was mainly driven by smaller RTs for the more ambiguous durations in the patient group. This demonstrated a specific form of decision deficit: the patients failed to slow down as controls in discriminating ambiguous durations. The RT difference between the most ambiguous duration (540 milliseconds) and the long standard (750 milliseconds) was negatively correlated with the disease severity score (UPDRS) in patients (Fig. 3C, *R* = −.46, *P* < .05). Therefore, the patients with more severe disease showed larger decision deficits as indexed by the RT difference between high and low ambiguous conditions.

We observed similar results from the trisection data. There was a significant main effect of duration (*F*_14,490_ = 11.58, *P* < .0001) and a significant duration by group interaction (*F*_14,490_ = 2.34, *P* < .01). Similar to the bisection task, the RTs in controls were larger in more ambiguous durations, whereas RTs in patients were faster than controls when the duration fell between the medium and long standards (Fig. 2D). Further analysis of the patient data showed similar trends of negative correlations between the UPDRS and the RT difference between the 2 most ambiguous durations (700 milliseconds and 750 milliseconds) and the medium standard (550 milliseconds), although the results did not reach significance (700‐milliseconds duration: *R* = −.41, *P* = .09, 750‐milliseconds duration: *R* = −.46, *P* = .05) (Fig. 3D).

It could be argued that the RT difference between PD patients and controls in time perception tasks is caused by simple impairments in the patients' motor speed. To exclude this possibility, we analyzed the RT in the baseline‐reaction‐time task. Patients could successfully perform the simple reaction‐time task (mean response rate 97.18%, 1.52% SEM) similar to the controls (mean response rate 98.95% ± 0.44%) with no significant difference in the mean response rate between groups (*F*_1,34_ = 1.38, *P* = .25). There was no significant difference in the RT of the baseline‐reaction‐time task between patients and controls (*F*_1,34_ = 1.96, *P* = .17), suggesting that our bisection and trisection results could not be adequately explained by potential impairments in making motor responses in patients.

### Computational Modeling of Temporal Bisection and Trisection

**Figure 4:**
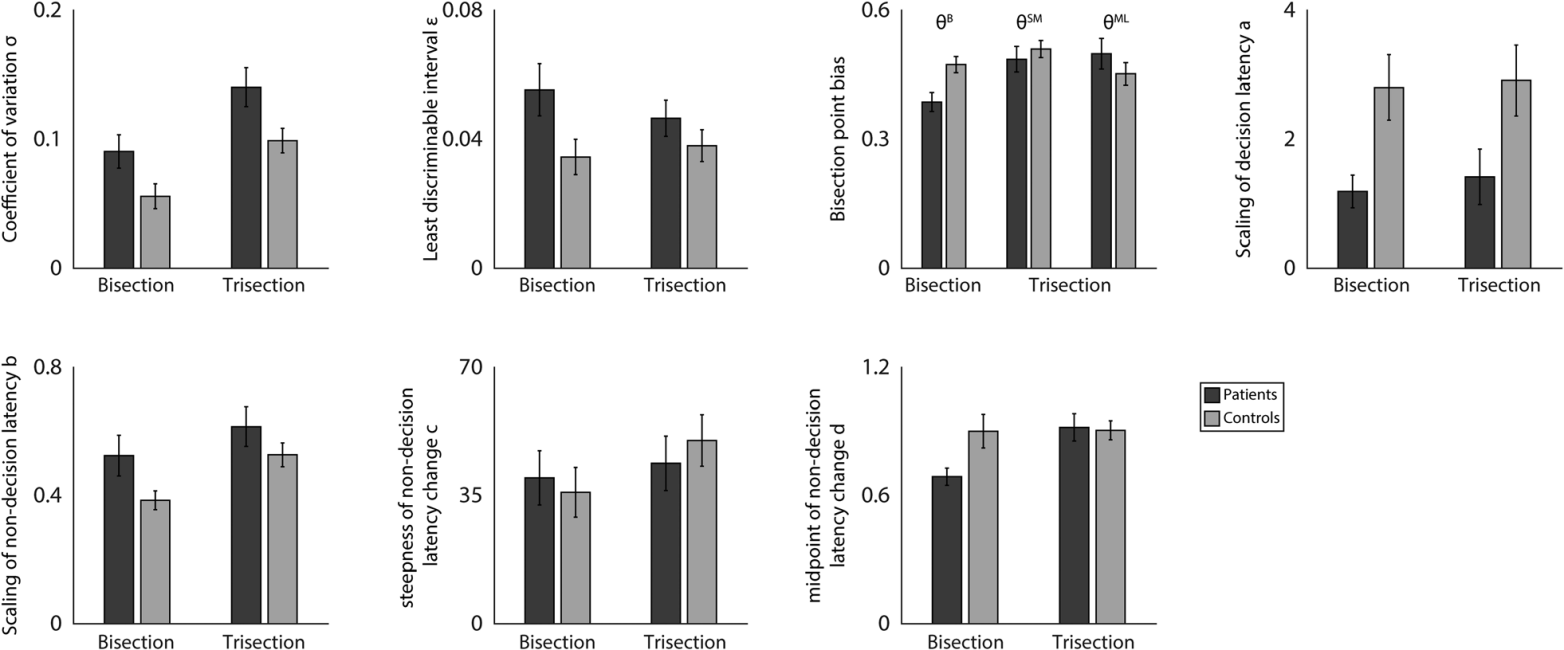
Model parameter values (see the Methods section for parameter definitions). Error bars denote standard errors of the mean across participants. Parameters *θ* ^*B*^, *θ* ^*SM*^, and *θ* ^*ML*^ refer to bisection point biases in the bisection task, trisection task at short‐medium duration, and trisection task at medium‐long duration, respectively.

The model provided an accurate quantitative prediction to the observed data (Fig. 2 and Supplementary Table 1). We compared the fitted model parameters between PD patients and controls (Fig. 4). For the coefficient of variation *σ*, a repeated‐measures ANOVA showed a main effect of group (*F*_1,35_ = 7.57, *P* < .05, FDR corrected), with higher values in patients in both tasks (bisection *P* < .05; trisection *P* < .05, permutation test). For the scaling parameter of decision latency *a*, there was a significant group difference (*F*_1,35_ = 8.18, *P* < .05, FDR corrected), with smaller values in patients in both tasks (bisection *P* < .01; trisection *P* < .05, permutation test). At a more liberal statistical threshold uncorrected for multiple comparison, patients had a larger least discriminable interval *ε* (*F*_1,35_ = 4.98, *P* < .05, uncorrected), and marginally larger scaling parameter for nondecision latency *b* (*F*_1,35_ = 4.03, *P = * .052, uncorrected). Therefore, when compared with controls, PD patients had a larger variability in representations of time intervals (lager *σ*) and shorter decision latency in ambiguous conditions (lower *a*). There was no significant group difference in other model parameters (*P > * .12, uncorrected). Across patients and controls, the trisection task had a larger coefficient of variation *σ* (*F*_1,35_ = 23.66, *P* < .001, FDR corrected) and larger scaling for nondecision latency *b* (*F*_1,35_ = 8.18, *P* < .001, FDR corrected) than the bisection task, suggesting that the trisection task is more cognitively demanding.

## Discussion

This is the first study to demonstrate the impairment of decision‐making in PD during interval timing. The behavioral results and computational modeling revealed both premature responding and disruption of timing sensitivity in PD. These impairments are associated with individual differences in dopaminergic therapy and disease severity.

PD patients had disproportionately faster RT than controls when making rapid decisions on more ambiguous intervals despite the presence of bradykinesia. The magnitude of this acceleration of RT increased with more advanced disease. This result is unlikely to be explained by patients' slowness of movement because they had a similar baseline response time as controls.

Previous research showed that similar deficits extends to other task domains, with faster RTs in PD in tasks of color discrimination,^38^ continuous performance,^48^ and discounting.^13^ Dopamine‐agonist treatment has been shown to increase this specific type of impulsive behavior.^13^ Most previous studies used trial‐by‐trial rewards as feedback. In the current study, the tasks did not include any monetary or other explicit incentives. Therefore, the accelerated RT in highly ambiguous conditions could be a generalized feature of the disease, independent of reward processing.

Premature responding during rapid decisions could be related to the notion that PD patients exhibit reflection impulsivity,^49^, ^50^ that is, a tendency to “jump to conclusions” without gathering sufficient information. Poor information sampling in PD has been examined explicitly in other impulsivity‐related tasks. For example, in a “bead” task,^51^ participants choose between waiting for further information and making inferences based on existing information.^52^ Similar to our findings, PD patients both with and without impulse‐control disorders made more impulsive and irrational choices based on less information than controls.^52^ It is worth noting that behavioral tasks that are specifically designed to measure such reflection impulsivity often focus on long time scales, such as the matching familiar figure task^50^ and the information sampling task.^49^ Here, we added to this emerging literature by suggesting that our data can be interpreted as a form of reflection impulsivity for perception tasks at a very short time scale.

An interesting finding is that premature responding positively correlates with disease severity. In a factor analysis of impulsivity construct in PD, multiple impulsivity factors positively correlate with clinical measures of disease severity (UPDRS),^12^ consistent with our results here. One possible cause is that, although dopaminergic replacement therapy restores motor function, nonmotor dopaminergic systems could be relatively overdosed,^10^, ^53^ leading to cognitive deficits such as choice impulsivity. Note, however, that not all PD‐related impulsivity is related to excess dopamine. For example, patients have impaired stop‐signal performance (motor inhibition) even before dopaminergic medication. The second possible cause is that premature responding associates with dysfunctions in other neurotransmitter systems in PD. For example, atomoxetine (selective noradrenaline reuptake inhibitor) has been shown to reduce reflection impulsivity^54^ as well as response disinhibition^55^, ^56^ in PD. Further pharmacological studies are needed to examine the relationship between changes in neurotransmitter systems and reflection impulsivity.

Our study provides new insights into timing impairments in PD. Given the compelling evidence for the central role of basal ganglia and dopaminergic projects in interval timing, timing deficits have been a long‐standing question on PD behavior. Previous studies have reported that patients are impaired with intervals lasting seconds to minutes^57^ and subsecond intervals,^58^ estimations of time intervals,^59^ and discrimination of interval durations.^30^ However, studies reporting no impairment in patients are abundant.^60^, ^61^, ^62^, ^63^, ^64^ A recent review of 37 time perception studies reported that only 62% of the studies show impaired performance in PD patients. This controversy may be partly a result of clinical heterogeneity in patient cohorts between studies^65^ as well as the range of tasks used.

The current study used a within‐subject design to minimize between‐patient variability on bisection and trisection tasks. The two tasks tested similar duration range (subsecond), but differed in their decision rules and cognitive demands. Although the current study does not directly indicate the neurobiological or neurochemical origins of the timing deficits in PD, we found that timing deficits do not fully generalize between the two tasks, highlighting the importance of task factors when assessing patients' performance.

It is important to note that the classical pacemaker model of time perception includes three independent information‐processing stages: clock, memory, and decision‐making.^22^, ^23^ Previous research largely focused on the abnormality of the clock and memory processes in PD. Here, computational modeling provided a unified account for behavioral performance in two different tasks. This integrated method made inferences on altered psychological processes in patients that are not readily available in raw behavioral measures, including decision deficits (scaling parameter to decision latency) as well as impaired precision of time representation (coefficient of variation).

Our modeling results suggested an increased variability of the mental representation of time intervals in patients. In other words, patients with PD have an impaired ability to generate reliable timing signal, which could contribute to the defining motor signs of bradykinesia, rigidity, and postural instability in PD.^15^ A recent study showed that 4 weeks training of rhythmic auditory cueing do not only improve gait in PD patients but also lead to beneficial effects on their perceptual and motor timing.^66^ Future studies are warranted to confirm whether restoring timing sensitivity, either through medical devices or medication, may have therapeutic effects on other motor and nonmotor symptoms in PD.

Two methodological limitations require further consideration. First, all of the patients in this study were tested when on their routine dopaminergic medications. Previous studies have compared timing performance between patients “on” and “off” dopaminergic medications, with inconsistent results. For example, detrimental performance with long time intervals (>10 seconds) was shown to be restored by administration of levodopa,^59^ whereas dopamine replacement therapy worsened performance in a time production task.^67^, ^68^ Given the differential neurodegeneration in separate cortico–striato–thalamo circuits, it is possible that the effects of PD and dopaminergic treatment on timing performance are not additive.^69^ Future pharmacology studies in combination with computation modeling would be necessary to delineate these effects.

Second, our model is developed from animal and human research on interval timing and naturally accounts for the scalar expectancy theory and Weber's law.^23^, ^70^ This model‐based approach goes beyond inferences about individual behavioral measures and provides an explanatory mechanism for the abnormal time perception. However, despite the adequate fit to individual data in the two tasks, it has yet to be determined how the psychological model is implemented in the brain. Nevertheless, our model can be formalized under an accumulation‐to‐threshold framework for perceptual decisions,^71^, ^72^, ^73^ for which the underlying neural mechanisms have been extensively studied.^74^, ^75^, ^76^ Recent studies indeed show promising results by using perceptual decision models to account for bisection performance.^77^, ^78^

In conclusion, we have demonstrated the presence of premature responding during time perception in PD. Patients with more advanced disease accelerated their responses when discriminating ambiguous intervals, and poor timing performance was associated with dopaminergic medication. These findings indicate a signature of reflection impulsivity that could also extend to longer time scales and be relevant to impulsive behavior in PD. The presence of both decision deficits and abnormal clock functions indicate the need for combined strategies to develop therapies for cognitive and behavioral impairment in PD.

## Author Roles

1. Research Project: A. Conception, B. Organization, C. Execution; 2. Statistical Analysis: A. Design, B. Execution, C. Review and Critique; 3. Manuscript Preparation: A. Writing the First Draft, B. Review and Critique.

J.Z.: 1A, 1B, 1C, 2A, 2B, 2C, 3A, 3B

C.N.: 1B, 1C, 3B

N.W.: 1B, 1C, 3B

R.B.: 1B, 3B

J.R.: 1B, 2C, 3A, 3B

## Full financial disclosures of all authors for the past 12 months

James Rowe received grants from Medical Research Council and Wellcome Trust.

Roger Barker received grants from National Institute of Health Research Biomedical Research Award.

## Supporting information

Additional Supporting Information may be found in the online version of this article at the publisher's web‐site.

Supporting Information
