# Supplementary material for: Time on timing: Dissociating premature responding from interval sensitivity in Parkinson's disease
Source: Mov Disord. 2016 Apr 19;31(8):1163–72. doi: 10.1002/mds.26631 (PMC4988382; doi:10.1002/mds.26631)
Supplement: Supplementary file 1 — Supporting Information [file MDS-31-1163-s001.doc]

**Time on timing: dissociating premature responding from interval sensitivity in Parkinson’s disease**

**Supplementary Material**

Zhang J, Nombela C, Wolpe N, Barker RA, Rowe JB

**Supplementary Table 1.** Drug details of PD patients. All doses are in milligrams. Other drugs are: C = COMT inhibitor, R = Rasagiline, S = Selegiline

| **No.** | **L-dopa** | **Pramipexole** | **Ropinirole** | **Other drugs** |
| --- | --- | --- | --- | --- |
| 1 | 800 | 3.15 |  | C |
| 2 | 600 |  | 16 |  |
| 3 | 1000 | 3.15 |  | C |
| 4 | 400 |  | 8 |  |
| 5 | 900 |  | 16 | C, R |
| 6 | 150 | 3.15 |  |  |
| 7 | 150 |  | 24 | R |
| 8 | 1050 | 1.05 |  |  |
| 9 | 1200 | 2.112 |  |  |
| 10 | 375 |  | 8 |  |
| 11 | 300 | 0.7 |  | S |
| 12 | 200 | 1.4 |  | C, S |
| 13 | 800 | 2.1 |  | C |
| 14 | 600 |  | 24 | R |
| 15 | 1125 | 3.15 |  | C |
| 16 | 1000 |  |  | R |
| 17 | 1000 |  |  | C |
| 18 | 600 |  |  | S |

**Supplementary Table 2.** Means and standard errors of bisection points and Weber ratios for Parkinson’s patients and controls in the bisection and trisection tasks. Model predictions were obtained by averaging the results from 100 simulations with the best fitted model parameters for each participant.

| **Task** | **Group** | **Data** | | **Model prediction** | |
| --- | --- | --- | --- | --- | --- |
|  |  | **Bisection points** | **Weber ratio** | **Bisection points** | **Weber ratio** |
| Bisection | Patients | 0.50 (0.01) | 0.18 (0.01) | 0.50 (0.01) | 0.19 (0.02) |
|  | Control | 0.53 (0.01) | 0.13 (0.01) | 0.53 (0.01) | 0.13 (0.01) |
| Trisection  (short-medium durations) | Patients | 0.38 (0.01) | 0.20 (0.01) | 0.37 (0.01) | 0.21 (0.02) |
|  | Control | 0.38 (0.01) | 0.17 (0.004) | 0.38 (0.01) | 0.17 (0.004) |
| Trisection (medium-long durations) | Patients | 0.72 (0.01) | 0.16 (0.02) | 0.73 (0.01) | 0.14 (0.01) |
|  | Control | 0.71 (0.01) | 0.13 (0.01) | 0.71 (0.01) | 0.12 (0.01) |


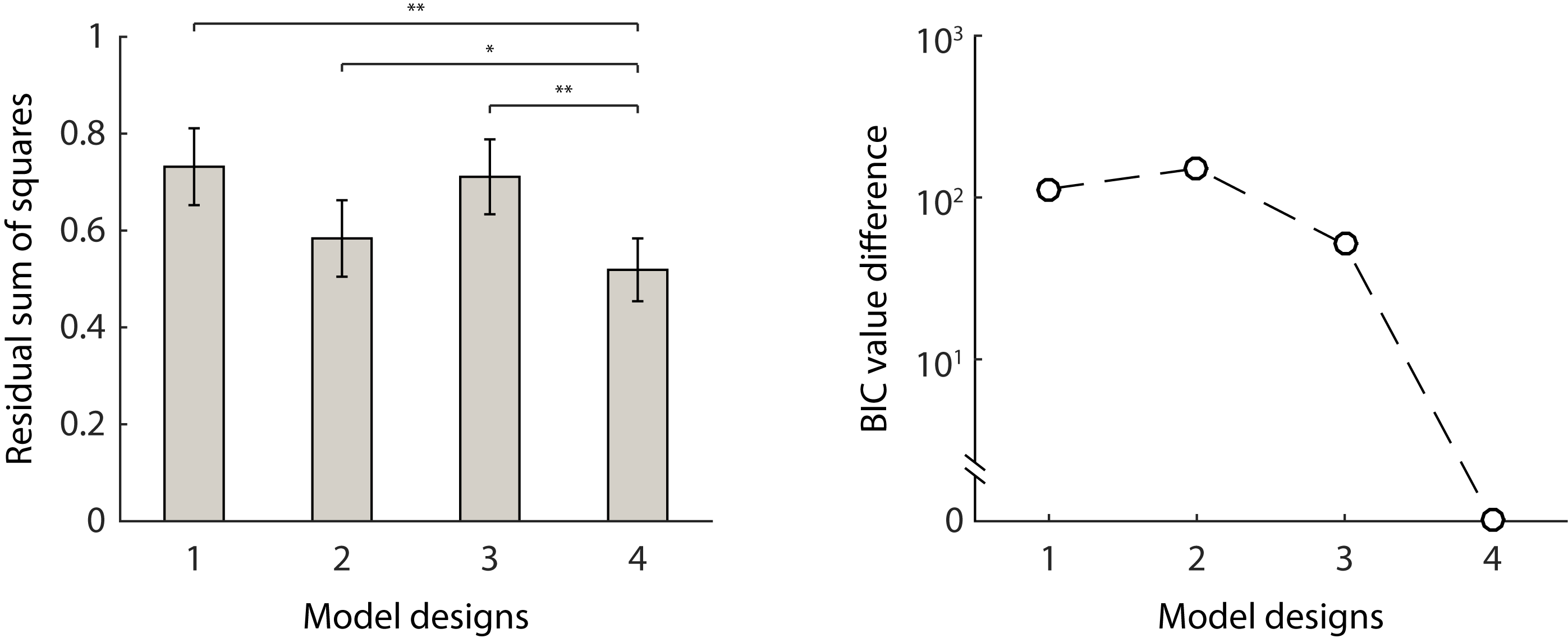


**Supplementary Figure 1.** Model comparison. (A) The mean residual sum of squares across participants for each model design. The error bars represent the standard errors across participants. Model 4 had significantly better fit (smaller residual errors) than Model 1 (*p*<0.001, Wilcoxon signed rank test), Model 2 (*p*<0.01), and Model 3 (*p*<0.001). (B) The BIC differences between the best fit model (Model 4) and the other three model variants. The best fit model assumed the scalar property of interval distributions and a nonlinear non-decision latency (see Supplementary Methods for parameter constraints of the other model designs).

Supplementary methods

Apparatus

Participants were seated in a sound-damped booth. Visual stimuli were presented on a LCD monitor (Dell 1908FP) with a resolution of 1024 × 768 and a refresh rate of 60 Hz, located about 50 cm in front of the participants. Auditory stimuli were presented diotically over a calibrated headphone (Sennheiser HD-280**,** Wedemark, Germany). Participants’ responses were collected from a keypad. The durations for time perception tasks were presented as 400 Hz auditory tones, produced by Matlab 7.8 (Mathworks, Natick, MA) and the Psychtoolbox-3 ([www.psychtoolbox.org](http://www.psychtoolbox.org/)).

Nonparametric permutation test

We used nonparametric permutation tests to assess the statistical significance of BP, WR and model parameters between groups, making no assumption about the normality of the data [1,2]. For example, to compare the BP between patients and controls, we calculated the observed test statistic as the sample t-value between the BP of patients and controls. We then generated a null distribution of the test statistic from 100,000 permutations with randomly shuffled data between groups in each permutation. A permutation *p*-value was obtained as the proportion of the permutation samples with the test statistic exceeded the observed value.

Computational modelling of data

To quantify an individual participant’s response as well as response time in both bisection and trisection tasks, we developed an extended version of the modified difference model of Wearden (1991) (Figure 1C). The model assumes that the subjective sense of a time interval *t* on trial *i* is randomly drawn from a Gaussian distribution with mean *t* (hereafter denoted by *u­_i_*(*t*). To account for the scalar property of time and Weber’s law [4,5], the standard deviation of the distribution equals *σt* (*σ*>0), which scales linearly with a constant coefficient of variation *σ*.

For the bisection task, the model draws three samples of durations on each trial: *u­_i_*(*S*), *u­_i_*(*L*) and *u­_i_*(*t*), where *u­_i_*(*S*), *u­_i_*(*L*) are the samples from the memory of the short and long standards, and *u­_i_*(*t*) is the sample of the presented duration. The bisection response is determined by the relative difference of the presented duration and the bisection point:

$$P_{i}(Long)=\left\{\begin{matrix} 0, for u_{i}(t)<BP_{i}^{B}-\frac{\varepsilon}{2} ("short" resposne), \\ 0.5, for BP_{i}^{B}-\frac{\varepsilon}{2}\leq u_{i}(t)\leq BP_{i}^{B}+\frac{\varepsilon}{2} ("short" or "long" resposne), \\ 1, for u_{i}(t)>BP_{i}^{B}+\frac{\varepsilon}{2} ("long" resposne), \end{matrix}\right.$$

where *P*(Long) is the probability of making the “long” response, and the subjective bisection point *BP^B^* on trial *i* is given by:

$$BP_{i}^{B}=\theta^{B}(u_{i}(L)-u_{i}(S))+u_{i}(S) .$$

The parameter *θ^B^* (0<*θ^B^*<1) determines the bisection bias of the model from the arithmetic mean (i.e., with *θ^B^*=0.5, the model predicts the bisection point is equivalent to the arithmetic mean). The value of *ε* represents the least discriminable interval of time. If the presented duration is close to the bisection point and within the least discrimination interval, the model makes a random decision between “short” and “long” responses.

The model can be easily extended to the trisection task, for which the model draws four samples of durations on each trial: *u­_i_*(*S*), *u­_i_*(*M*), *u­_i_*(*L*) and *u­_i_*(*t*), where *u­_i_*(*S*), *u­_i_*(*M*), *u­_i_*(*L*) are the samples from the memory of the short, medium and long standards. The trisection response is determined by the relative difference of the presented duration and two bisection points: one for the short-medium range and the other for the medium-long range. The probability of “medium” response is given by:

$$P_{i}(Medium)=\left\{\begin{matrix} 0, for u_{i}(t)<BP_{i}^{SM}-\frac{\varepsilon}{2} ("short" resposne), \\ 0.5, for BP_{i}^{SM}-\frac{\varepsilon}{2}\leq u_{i}(t)\leq BP_{i}^{SM}+\frac{\varepsilon}{2} ("short" or "medium" resposne), \\ 1, for BP_{i}^{SM}+\frac{\varepsilon}{2}<u_{i}(t)<BP_{i}^{ML}-\frac{\varepsilon}{2} ("medium" resposne), \\ 0.5, for BP_{i}^{ML}-\frac{\varepsilon}{2}\leq u_{i}(t)\leq BP_{i}^{ML}+\frac{\varepsilon}{2} ("medium" or "long" resposne), \\ 0, for u_{i}(t)>BP_{i}^{ML}+\frac{\varepsilon}{2} ("long" resposne), \end{matrix}\right.$$

where the bisection points *BP^SM^* (for the short-medium range) and *BP^ML^* (for the medium-long range) are given by:

$$\begin{matrix} BP_{i}^{SM}=\theta^{SM}(u_{i}(S)-u_{i}(M))+u_{i}(S) , \\ BP_{i}^{ML}=\theta^{ML}(u_{i}(M)-u_{i}(L))+u_{i}(M) . \end{matrix}$$

The model further assumes that the observed response time in both bisection and trisection tasks is determined by the latencies of a decision process and a non-decision process. The decision latency is associated with the ambiguity of the presented duration, which is quantified by the distance between the presented duration and the standard duration at which the response is being made. When the presented duration is closer to a standard duration, the stimuli is less ambiguous and easier for categorization, leading to shorter decision time. This account is consistent with a recent model that applies the sequential sampling theory to time discrimination performance [6]. The non-decision latency is used to describe stimulus encoding and motor response delays [7]. We expected the non-decision latency remains constant for short presented durations, and decreases as a function of the presented duration when the elapsed time in a trial exceeds a certain threshold. The rationale is that when the presented duration is long enough, a “long” response could be committed prior to the offset of the presented duration, and the non-decision component in the response time could be reduced by beginning a motor preparation before stimulus offset (for in-depth discussion on this issue, see Balci & Simen, 2014; Çoşkun, Sayalı, Gürbüz, & Balcı, 2014). Specifically, the model prediction of RT is given by a weighted sum of decision and non-decision latencies:

$$RT(i)=a\left|u_{i}(t)-u_{i}(R)\right|+b\frac{1}{1+e^{-c(d-u_{i}(t))}} ,$$

where *u_i_*(*R*) is the sampled value of the standard duration corresponding to the model’s response in trial *i* (*R*=[*S*, *L*] in bisection, and *R*=[*S*, *M*, *L*] in trisection). The value of *a* determines how decision latencies scale with stimulus ambiguity. The non-decision latency was parameterized by a logistic function, where *b* is the maximum non-decision latency, *c* represents the steepness of the change in non-decision latency, and *d* is the threshold duration at which the non-decision latency decreases to 50% of its maximum.

We fitted the model to individual behavioural data (mean response and mean response times at each of the presented durations), separately for the bisection and trisection tasks. The model prediction of behavioural performance for each condition was estimated from 10,000 simulations. There are seven parameters for the bisection task (*σ*, *ε*, *θ^B^*, *a*, *b*, *c*, and *d*) and eight parameters for the trisection task (*σ*, *ε*, *θ^SM^*, *θ^ML^*, *a*, *b*, *c*, and *d*). The model parameters were determined by minimizing the sum of the squares of the errors between the data and model predictions using the simplex algorithm in Matlab [9]. For each measure of choice or RT, the error between observed data and model prediction was normalized before calculating the overall model errors. The normalization factor for each statistic was taken as the mean of the observed data for its type (choice or RT). This procedure ensures that RT and choice contribute equally to the overall model error. To avoid settling in a local minimum during parameter optimization, the optimization procedure was repeated with 40 iterations, and for each time started with a different set of initial parameters chosen from 100 random parameter samples which produced the best fit. This method combined coarse search with fine search over parameter space, which has been successfully used for parameterization of psychological models [10]. The best-fitting parameters from the 40 iterations were then used to calculate the model prediction of behavioural performance for each participant.

Comparison with other candidate models

Three other candidate models were considered. The total of four variants of the model differed in their parameter constraints. The first model assumed a constant standard deviation of interval distributions (i.e., violation of the scalar property) and a constant non-decision latency. The second model assumed violation of the scalar property and a nonlinear non-decision latency (a logistic function). The third model assumed the scalar property of interval distributions and a constant non-decision latency. The fourth model assumed the scalar property and a nonlinear non-decision latency (i.e., the model described above).

For each model, we fitted individual behavioural data by using the same least-squares optimization procedure. In order to assess model adequacy, the best-fitting parameters of each model were used to calculate the residual sum of squares for each participant’s data and the overall Bayesian information criterion (BIC) across participants (Supplementary Figure 2).

References

1 Edgington E. *Randomization Tests, Fourth Edition*. Taylor & Francis 1995.

2 Coolican H. *Research Methods and Statistics in Psychology, Fifth Edition*. London: : Hodder Education 2009.

3 Wearden JH. Human performance on an analogue of an interval bisection task. *The Quarterly journal of experimental psychology B, Comparative and physiological psychology* 1991;**43**:59–81.

4 Merchant H, Luciana M, Hooper C, Majestic S, Tuite P. Interval timing and Parkinson’s disease: Heterogeneity in temporal performance. *Experimental Brain Research* 2008;**184**:233–48.

5 Merchant H, Zarco W, Prado L. Do we have a common mechanism for measuring time in the hundreds of millisecond range? Evidence from multiple-interval timing tasks. *Journal of neurophysiology* 2008;**99**:939–49.

6 Balci F, Simen P. Decision processes in temporal discrimination. Acta Psychol. (Amst). 2014.

7 Ratcliff R, McKoon G. The diffusion decision model: theory and data for two-choice decision tasks. *Neural computation* 2008;**20**:873–922.

8 Çoşkun F, Sayalı ZC, Gürbüz E, Balcı F. Optimal time discrimination. *The Quarterly Journal of Experimental Psychology* 2014;**68**:381–401.

9 Nelder JA, Mead R. A simplex method for function minimization. *The Computer Journal* 1964;**7**:308–13.

10 Bogacz R, Cohen JD. Parameterization of connectionist models. *Behavior research methods, instruments, & computers : a journal of the Psychonomic Society, Inc* 2004;**36**:732–41.
